# Statistical Estimation of Correlated Genome Associations to a Quantitative Trait Network

**DOI:** 10.1371/journal.pgen.1000587

**Published:** 2009-08-14

**Authors:** Seyoung Kim, Eric P. Xing

**Affiliations:** School of Computer Science, Carnegie Mellon University, Pittsburgh, Pennsylvania, United States of America; Princeton University, United States of America

## Abstract

Many complex disease syndromes, such as asthma, consist of a large number of highly related, rather than independent, clinical or molecular phenotypes. This raises a new technical challenge in identifying genetic variations associated simultaneously with correlated traits. In this study, we propose a new statistical framework called graph-guided fused lasso (GFlasso) to directly and effectively incorporate the correlation structure of multiple quantitative traits such as clinical metrics and gene expressions in association analysis. Our approach represents correlation information explicitly among the quantitative traits as a quantitative trait network (QTN) and then leverages this network to encode structured regularization functions in a multivariate regression model over the genotypes and traits. The result is that the genetic markers that jointly influence subgroups of highly correlated traits can be detected jointly with high sensitivity and specificity. While most of the traditional methods examined each phenotype independently and combined the results afterwards, our approach analyzes all of the traits jointly in a single statistical framework. This allows our method to borrow information across correlated phenotypes to discover the genetic markers that perturb a subset of the correlated traits synergistically. Using simulated datasets based on the HapMap consortium and an asthma dataset, we compared the performance of our method with other methods based on single-marker analysis and regression-based methods that do not use any of the relational information in the traits. We found that our method showed an increased power in detecting causal variants affecting correlated traits. Our results showed that, when correlation patterns among traits in a QTN are considered explicitly and directly during a structured multivariate genome association analysis using our proposed methods, the power of detecting true causal SNPs with possibly pleiotropic effects increased significantly without compromising performance on non-pleiotropic SNPs.

## Author Summary

An association study examines a phenotype against genotypic variations over a large set of individuals in order to find the genetic variant that gives rise to the variation in the phenotype. Many complex disease syndromes consist of a large number of highly related clinical phenotypes, and the patient cohorts are routinely surveyed with a large number of traits, such as hundreds of clinical phenotypes and genome-wide profiling of thousands of gene expressions, many of which are correlated. However, most of the conventional approaches for association mapping or eQTL analysis consider a single phenotype at a time instead of taking advantage of the relatedness of traits by analyzing them jointly. Assuming that a group of tightly correlated traits may share a common genetic basis, in this paper, we present a new framework for association analysis that searches for genetic variations influencing a group of correlated traits. We explicitly represent the correlation information in multiple quantitative traits as a quantitative trait network and directly incorporate this network information to scan the genome for association. Our results on simulated and asthma data show that our approach has a significant advantage in detecting associations when a genetic marker perturbs synergistically a group of traits.

## Introduction

Many complex disease syndromes, such as diabetes, asthma, and cancer, consist of a large number of highly related, rather than independent, clinical phenotypes. Differences between these syndromes involve a complex interplay of a large number of genomic variations that perturb the function of disease-related genes in the context of a regulatory network, rather than each gene individually [1],[2]. Thus, unraveling the causal genetic variations and understanding the mechanisms of consequent cell and tissue transformation requires an analysis that jointly considers the epistatic, pleiotropic, and plastic interactions of elements and modules within and between the genome, transcriptome, and phenome. Until now, most popular approaches for genetic and molecular analysis of diseases were mainly based on classical statistical techniques, such as the linkage analysis of selected markers [3],[4]; quantitative trait locus (QTL) mapping [5],[6] conducted over one phenotype and one marker genotype at a time, which are then corrected for multiple hypothesis testing [7],[8]; and primitive data mining methods, such as the clustering of gene expressions and the high-level descriptive analysis of molecular networks. Such approaches yield crude, usually qualitative characterizations of the study subjects.

Numerous recent studies have shown that it is often more informative to map intermediate steps in disease processes, such as various disease-related clinical traits or expression levels of genes of interest, rather than merely the binary case/control disease status, to genetic marker loci [2], [9]–[13]. These molecular and clinical traits provide detailed insight to the relationship between genome variations and disease phenotypes because they are more directly influenced by the genotype variations. Furthermore, since many of these intermediate traits in a complex multivariate phenotype are highly correlated, combining information across multiple such traits during the analysis of genome-phenome association can offer a deeper insight on the possibly multi-factorial functional roles that the associated genotype variations may play to give rise to the disease under study. At the same time, they can provide a greater power for detecting weak association signals that might have been missed if each trait was analyzed separately.

**Figure 1:**
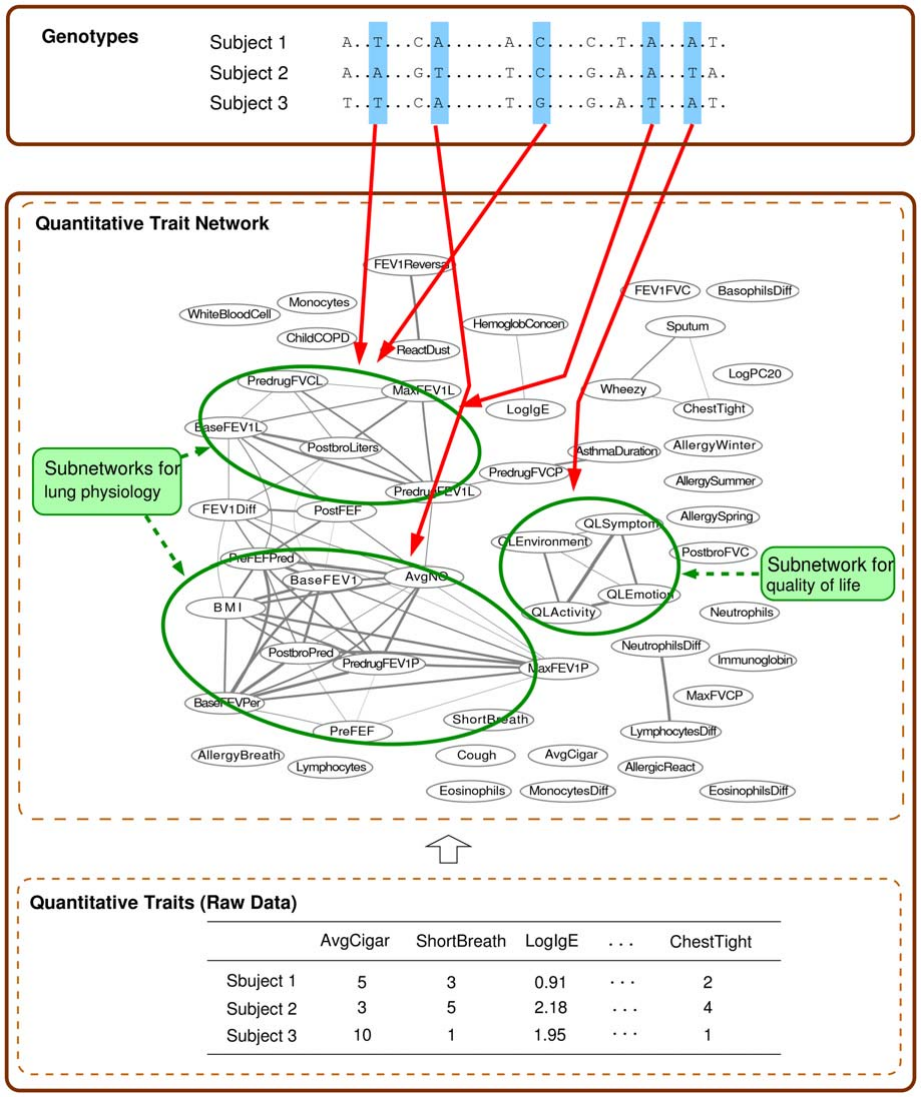
An illustration of association analysis using the QTN for asthma dataset. Nodes in the QTN represent clinical traits related to asthma. Each pair of nodes is connected with an edge if the corresponding two traits are highly correlated. The thicknesses of edges indicate the strength of correlation. We are interested in identifying SNPs that are associated with a subnetwork of clinical traits.

In several recent attempts on expression quantitative trait locus (eQTL) mapping, a significant focus has been placed on identifying modules of co-expressed genes and the genotype markers that perturb the whole module rather than a single gene. For example, a genotype variation in a putative transcription factor is likely to affect the expression levels of all of the genes regulated by this common transcription factor. Under this scenario, once a group of genes are mapped to a common locus in the genome, it is possible to examine whether the locus harbors a transcription factor that targets the group of genes jointly in order to understand the functional relationship between the genotype marker and the gene module (e.g., [11]). Another example, which will be explored in this paper, involves the study of complex heterogeneous diseases such as asthma that cannot be characterized by a single phenotype, but are influenced by multiple factors. In Figure 1, the correlation structure of 53 clinical traits in an asthma dataset collected as a part of the Severe Asthma Research Program (SARP) [14] is represented as a *quantitative trait network* (QTN). From a visual inspection of this network, it is apparent that it contains several groups of inter-correlated traits that are connected with weighted edges among them. Further investigation reveals that each subnetwork in this QTN corresponds to different clinical aspects of asthma, such as quality of life (the nodes for QLEnvironment, QLSymptom, QLEmotion, and QLActivity), asthma symptoms (the nodes for Wheezy, Sputum, ChestTight), and lung physiology (the nodes for BaseFEV1, PreFEFPred, PostbroPred, PredrugFEV1P, MaxFEV1P, etc.). It is natural for one to suspect that such highly correlated traits in a subnetwork may share some common genetic causes, and that analyzing a group of traits in each subnetwork jointly rather than each trait independently may help to better uncover such causes.

Recent advances in high-throughput sequencing and molecular profiling technologies have made it both affordable and efficient to observe DNA sequence variations over millions of genomic loci, to measure the abundance of transcripts of virtually all known coding sequences, and to measure a wide range of clinical traits in various disease populations [5],[6],[15],[16]. As more phenotype data are available at a phenome scale, one immediate methodological challenge arising in the analysis is how to detect joint associations between a polymorphic marker to a phenome of multiple correlated traits. Indeed, there has been a lack of statistical tools for a joint analysis of multivariate traits, related via a QTN, in a principled manner. In QTL mapping studies with pedigree data, a principal component analysis (PCA) has been applied to extract the components that explain the majority of the variation among traits, and a single-trait association analysis has been performed on each of the transformed trait separately [17],[18]. However, this approach involves only an indirect form of structural information present in the traits, and has a limitation in that it is not obvious how to interpret the derived phenotypes. In several previous studies that incorporated a gene co-regulation network in a genome-wide scan for associations [2],[10],[11], a heuristic procedure was employed that combines results from two separate analyses, one being traditional single-SNP/single-trait association tests and the other being an *ad hoc* cluster analysis for finding gene modules from the co-regulation network. Subsequently, each cluster would undergo an examination to determine whether it contains a significantly large fraction of genes that are mapped to a common locus in the genome with a potential pleiotropic effect. This primitive approach is essentially a multitude of single-marker/single-trait analyses which involved no direct integration of information across traits within a QTN during the association tests themselves, since the clustering information was used only in the post-processing step.

In a different approach to eQTL mapping, a module network [19], which is a statistical model developed for uncovering regulatory modules from gene expression data, was extended to incorporate genotype information such that the expression levels of genes regulated by the same regulator are explained by the variations in both the expression levels of regulators and the genotypes of markers in question [9],[20]. This method estimated modules and associations jointly by iterating between learning gene modules through clustering and learning associations (i.e., which genes and markers regulate the module). The expression levels of genes in each module were summarized as an average of the members within the module, and then this “average phenotype” was mapped to genotypes and expression levels of other genes. However, using an averaged value over traits in a module can lead to a significant loss of information. For example, two genes in the same module might be negatively correlated in their responses to the common regulators, and an average of the two genes would conceal their individual associations to the common regulators. Thus, this method is not able to capture detailed relationships among multiple correlated traits such as the asthma QTN in Figure 1.

We believe that explicitly incorporating the molecular and/or clinical phenotype network as a trait correlation structure while searching for genetic associations can significantly increase the power of detecting pleiotropic effects. In this article, we present a new statistical approach, called *graph-guided fused lasso* (GFlasso), that can effectively address the general problem of association mapping of multivariate traits related as a quantitative trait network. Instead of using a two-stage method that performs single-trait analyses and combines the results afterwards in light of clusters of traits, our method directly infers markers with a pleiotropic effect by combining information across multiple traits in a single statistical framework, and does not require subnetworks or trait clusters to be extracted *a priori* or at any point of running the algorithm. The proposed GFlasso approach represents the correlation pattern in multiple traits explicitly as a QTN, and searches for genotype markers that are significantly and jointly associated with multiple highly correlated traits that often appear as a densely connected subnetwork within the whole network. Indeed, the extent of the “jointness” in a marker-to-multitrait association is automatically determined by the connectivities among traits in the QTN, and is subject to the modulation of the strengths of the trait correlations. Thus, the clustering information is just one form of relationship implicitly captured in the network, as the QTN is strictly richer than a trait-cluster. In addition, a QTN may carry other relational information such as weak correlations, heterogeneous (e.g., positive or negative) correlations, and pathways, etc. For example, the QTN of asthma-related traits in Figure 1 contains a large subgraph on the left which again contains two groups of densely connected traits. This hierarchical grouping information will be lost if we simply apply a clustering algorithm.

**Figure 2:**
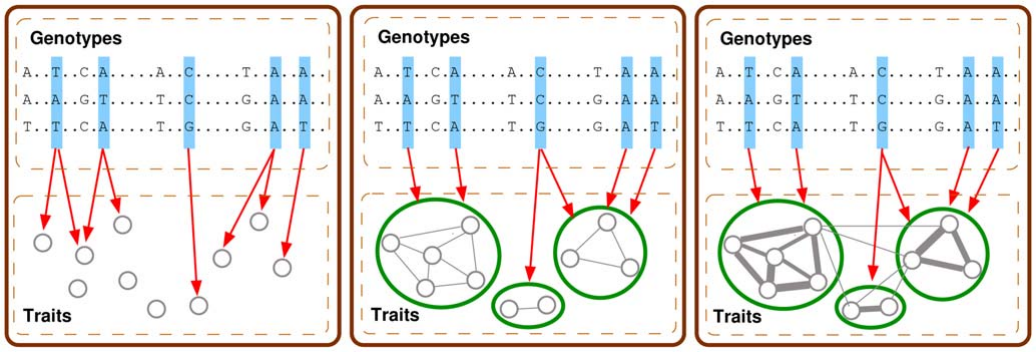
Illustrations for association analysis with multiple quantitative traits using various regression methods. (A) In lasso, each phenotype represented as a circle is independently mapped to SNPs for association. (B) In graph-constrained fused lasso (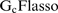), we consider a QTN to search for an association between a SNP and a subnetwork of traits. (C) In graph-weighted fused lasso (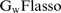), we consider a QTN with edge weights.

Our proposed approach is based on a regularized multivariate regression formalism, treating genotype markers as inputs and traits as outputs. To ensure interpretable and consistent recovery of the usually “sparse” causal (or “truly” relevant) variations among a large number of candidate polymorphic loci, we use a linear regression formalism with an 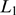 penalty, commonly known as *lasso*. Lasso achieves “sparsistancy” in the estimated model by setting the regression coefficients for irrelevant markers to exactly zero [21],[22]. As a brief digression for clarity, sparsistancy refers to an asymptotic property in high-dimensional statistical inference that for an estimator of a 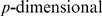 vector 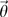 from 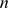 independent and identically-distributed samples, where 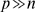, the probability of recovering the true non-zero elements 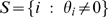 in the estimator approaches one in the limit, if the true non-zero elements are sparse in the sense that 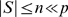 [22]. This property of lasso makes it a natural approach for genome-wide association analysis, where the (sparse) set of markers having non-zero regression coefficients are interpreted as the markers truly associated with the phenotype. However, when applied to an association mapping with multivariate traits, lasso is equivalent to a single-trait analysis that needs to be repeated over every single trait [23]. In other words, for a collection of possibly related traits, each trait would be treated as independent of all of the other traits, and regressed on a common set of marker genotypes via its own lasso (Figure 2A), ignoring the possible coupling among traits. Our innovation in GFlasso that enables a departure from the baseline lasso for a single trait is that, in addition to the lasso penalty, we employ a “fusion penalty” that fuses regression coefficients across correlated phenotypes, using either unweighted or weighted connectivity among individual traits in the QTN as a guide. This additional penalty will introduce soft constraints on the regression coefficients from the same genomic locus to different traits connected in the QTN, encouraging sharing of common predictors (i.e., associated markers) among coupled responses (i.e., traits). The two different choices of the fusion scheme lead to two variants of GFlasso: *graph-constrained fused lasso* (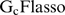) based on the constraints induced only by the QTN topology (Figure 2B), and *graph-weighted fused lasso* (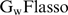) based on constraints with a flexible range of stringency determined by the edge weights in the QTN (Figure 2C). In this article, we are mainly concerned with continuous-valued traits, but the method can be extended to include a logistic regression model for discrete-valued traits.

The problem of estimating the regression coefficients in GFlasso involves solving a convex program, in which a global optimum solution can be efficiently obtained by exploring the large body of existing work on fast algorithms for convex optimization. In this article, we develop a fast coordinate-descent algorithm to estimate the regression coefficients under GFlasso, from which markers relevant to the (possibly multiple) traits in questions can be identified from the non-zero elements in the estimated regression coefficients. The results on two datasets, one simulated from HapMap SNP markers and the other collected from the SARP asthma patients, show that our method has a significantly greater power with fewer false positives in detecting pleiotropic effects of markers than other methods that do not exploit the correlation structure in traits.

## Methods

To capture correlated genome associations to a QTN, we employ a multivariate linear regression model as the basic model for trait responses given inputs of genome variations such as SNPs, with the addition of a sparsity-biasing regularizer to encourage selection of truly relevant SNPs in the presence of many irrelevant ones. Then, we introduce an additional regularizer of fusion penalty to encourage the sharing of association patterns from a common SNP to multiple inter-related traits.

There is a large literature on multivariate linear regression in statistics [24], and this approach has been previously applied to association analysis [23],[25]. However, earlier attempts have been solely focused on uncorrelated trait analysis. To establish a natural connection between our proposed methods and these earlier works, and to layout the necessary notations in our formulation, we start our presentation with an introduction to the standard regularized multivariate regression, which treats each trait as independent of the other traits. Then, we extend this formulation to exploit the correlation structure in multiple quantitative traits represented as a QTN.

### Lasso Regression for Multiple Independent Traits

In a standard regression approach for a single-trait association analysis, we assume a linear relationship between the covariates (SNPs) and each response (trait) parameterized by a set of regression coefficients, and estimate the parameters by optimizing a loss function defined on SNP-trait samples given the parameters. Based on the magnitudes of estimated regression coefficients, we draw conclusions on which SNPs are most significantly associated with the given trait. When data are available for multiple traits, we can apply this single-trait approach to each trait separately as we detail below.

Let 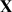 be an 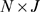 design matrix of genotypes for 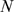 individuals and 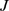 SNPs, where each element 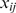 of 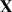 is assigned 0, 1, or 2 according to the number of minor alleles at the 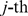 locus of the 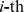 individual. Let 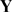 denote an 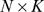 matrix of 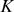 quantitative-trait measurements over the same set of individuals. We use 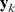 to denote the 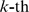 column (i.e., the 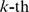 trait) of 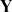. A conventional single-trait association via linear regression model can be applied to this multiple-trait setting by fitting the model to 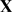 and each of the 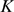 traits 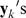 separately:
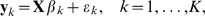
(1)where 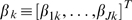 is a column vector of regression coefficients for the 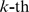 trait that can be used in a statistical test to detect SNP markers with a significant association, and 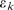 is a column vector of 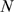 independent error terms with mean 0 and a constant variance. We center each column of 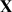 and 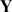 such that 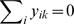 and 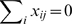, and consider the model in Eqn 1 without an intercept. Then, an estimate of 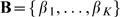 can be obtained by minimizing the residual sum of squares:
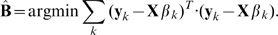
(2)The set of SNPs associated with the 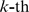 trait can be uncovered from the non-zero elements of the estimated coefficient vector 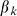, i.e., 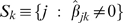.

In a typical genome-wide association mapping, one examines a large number of marker loci with the goal of identifying only a small number of markers associated with the given phenotype. A naive application of the method in Eqn 2 to association mapping with large 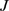 can cause several problems such as an unstable estimate of regression coefficients and a poor interpretability of 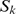 due to many irrelevant markers with non-zero regression coefficients. In order to handle the situation with large 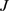, sparse regression methods such as forward stepwise selection [26], ridge regression [25],[27], and lasso [21] have been proposed. The main idea behind these methods is to select a relatively small subset of markers (or covariates) as associated with the trait, and set the regression coefficients for the rest of the markers to zero. Forward stepwise selection method iteratively selects one relevant marker at a time while trying to improve the model fit based on Eqn 2. However, it may not produce an optimal solution because of the greedy nature of the algorithm. A different approach based on regularization performs the selection in a continuous space by penalizing the residual sum of squares in Eqn 2 with an 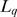 norm (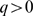) of 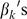 and shrinking the regression coefficients toward zero. For example, ridge regression is one such method that uses an 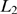 norm. However, it only shrinks the regression coefficients for irrelevant markers toward zero, and does not set them exactly to zero. We use lasso that employs an 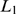 norm as a penalty because it has the property of setting the parameters for irrelevant markers exactly to zero. The lasso estimate of the regression coefficients can be obtained by solving the following 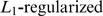 linear regression:
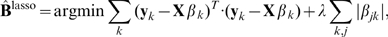
(3)where 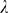 is a regularization parameter that controls the amount of sparsity in the estimated regression coefficients. Setting 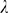 to a large value increases the amount of penalization, setting more regression coefficients to zero. Several efficient algorithms are available for solving the optimization problem defined by Eqn 3 [21],[28].

The lasso for multiple-trait association mapping defined in Eqn 3 is equivalent to solving a set of 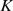 independent regressions for each trait with its own 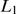 penalty. In other words, it does not provide any mechanism to combine information across multiple traits such that the estimates 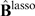 reflect the potential relatedness in the regression coefficients for those correlated traits in the QTN that can be potentially influenced by common SNPs. Below, we extend the standard lasso and propose new penalized regression methods for detecting markers with pleiotropic effect on correlated quantitative traits.

### Graph-Guided Fused Lasso for Multiple Correlated Traits

In order to estimate the association strengths jointly for multiple correlated traits while maintaining sparsity, we introduce another penalty term called graph-guided fusion penalty into the lasso framework. This novel penalty makes use of the complex correlation pattern among the traits represented as a QTN, and encourages the traits which appear highly correlated in the QTN to be influenced by a common set of genetic markers. Thus, the GFlasso estimate of the regression coefficients reveals joint associations of each SNP with the correlated traits in the entire subnetwork as well as associations with each individual trait.

We assume that a QTN, denoted by 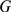, with a set of nodes 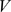 and a set of edges 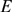 is available from a pre-processing step. Each edge 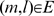 in QTN 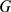 is associated with a weight that corresponds to some measures of strength of the correlation between the two nodes connected by the edge. In this article, we adopt a simple and commonly-used approach for inferring a QTN from data, where we first compute pairwise Pearson correlation coefficients for all pairs of phenotypes using 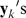, and then connect two nodes with an edge if their correlation coefficient is above a given threshold 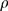. The weight of each edge 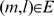 is set to the absolute value of the correlation coefficient, 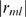. This thresholded correlation graph is also known as a *relevance network*, and has been widely used as a representation of gene interaction networks [29],[30]. Other variations of the standard relevance network have been suggested [31], and any of these QTNs as well as various other methods for learning a QTN can also be used within our proposed regression methods. The inference of a QTN and the definition of a node-correlation score are left as a user-specified option, and therefore, are not the main focus in this paper.

Below, we first introduce 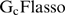 that makes use of only the information of graph topology, and then, further extend this method to 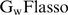 to take into account the full information in the QTN including edge weights.

### Model I: 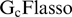


Given a QTN, it is reasonable to assume that if two traits are highly correlated and connected with an edge in the QTN, their variations across individuals are more likely to be explained by genetic variations at the same loci. In 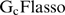, this bias is encoded as an additional penalty term that encourages a fusion of two regression coefficients 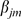 and 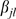 for each SNP marker 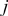 if traits 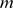 and 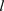 are connected with an edge in the QTN, as follows:
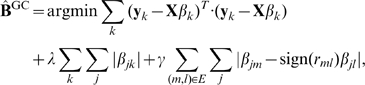
(4)where 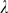 and 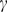 denote the regularization parameters that determine the amount of penalization from sparsity and fusion, respectively. The last term in Eqn 4 is called a fusion penalty [32], also known as a total variation cost in other contexts, and encourages (but does not strictly enforce) 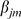 and 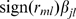 to take the same value by shrinking the difference between them toward zero. As a result, the fusion penalty tends to flatten the values of regression coefficients for each marker across multiple highly correlated phenotypes, so that the strength of influence of each marker becomes similar across those traits. We assume that if two traits 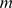 and 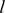 connected with an edge in 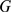 are negatively correlated with 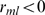, the effect of each marker on those traits takes an opposite direction, and we fuse 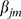 and 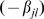, or equivalently, 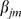 and 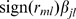. A larger value for 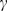 leads to a greater fusion effect, or greater sparsity in 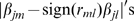.

The idea of using a fusion penalty has been first proposed in the classical regression problem for a univariate response (i.e., single output) and high-dimensional covariates to fuse the regression coefficients of two adjacent covariates when the covariates are assumed to be ordered such as in time [32]. This corresponds to coupling pairs of elements in the adjacent rows of the same column in the 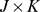 coefficient matrix 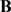 in Eqn 4. In 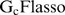, we employ a similar strategy in a multiple-output regression in order to identify pleiotropic effect of markers. Now, we let the QTN determine which pairs of regression coefficients should be fused, and for each edge, fuse every such coupled coefficient pair that corresponds to the elements of the corresponding two columns in the same row of matrix 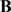 in Eqn 4. It is possible to show the asymptotic properties of estimators of the GFlasso methods as 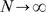 analogous to the ones previously shown for lasso and fused lasso [32],[33]. Recall that in genetic association mapping, our main goal is to recover the set of SNPs that are truly relevant to the traits in question, rather than the strengths of the associations captured by the magnitudes of elements in 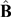. Thus, for the 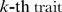 trait, the set of associated SNPs can be recovered from 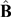 as 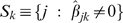.

When applied locally to a pair of regression coefficients for each edge 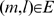 in the QTN, the fusion penalty can combine information across the two correlated traits for the given edge to potentially increase power for detecting true associations while reducing false positives. For example, if two traits connected by an edge in the QTN are only weakly affected by a common SNP, the fusion penalty for the corresponding edge can combine the two weak signals, and detect the associations that might have been missed under a single-trait analysis. Similarly, the information of a SNP being irrelevant is combined across two correlated traits connected with an edge, and both of the two regression coefficients for the irrelevant SNP are fused to zero, resulting in fewer false positives.

When this edge-level fusion penalty is applied to all of the edges in the entire QTN as in the graph-guided fusion penalty, the overall effect is that 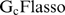 discovers associations between a SNP and a phenome as well as associations between a SNP and a single phenotype. This is because for each edge in the QTN, the fusion effect propagates through the neighboring edges, fusing the regression coefficients for each pair of traits connected by an edge, where the amount of such propagation is determined by the level of local edge connectivities. For example, within the subnetwork of densely connected traits that form a phenome, the fusion is effectively applied to all of its member traits, leading to an association with the phenome. On the other hand, if the edge connections are sparse within a subset of nodes in the QTN because of weak correlations among them, there will be little propagation of the fusion effect through the edges in the subgroup. As we demonstrate in the experiments, in the GFlasso estimate of 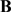, the set of non-zero regression coefficients tends to show a block structure with the same or similar values across correlated traits (or a phenome) for each genotype marker. Unlike other previous approaches for detecting the pleiotropic effect, which usually first apply some clustering algorithms to learn subgroups of traits and then search for genetic variations that perturb each subgroup, 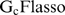 uses the full information on the correlation structure in the QTN, where the subgroup information is embedded implicitly within the QTN as densely connected subgraphs. Thus, 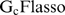 incorporates the subgrouping information from the QTN in a more flexible manner compared to previous approaches based on a clustering algorithm.

Although, in principle, the graph-guided fusion penalty has a smoothing effect on the rows of 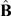, and encourages similar magnitudes of the association strengths from a given SNP to traits within a densely connected subgraph, the application of 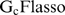 and other GFlasso methods described in the sequel does not strictly require the association strengths of each SNP to be identical across all correlated traits in the observed data. We emphasize that the 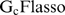 penalty introduces a bias favoring closeness in the magnitudes of the regression coefficients for correlated traits rather than enforcing a hard constraint that the fused regression coefficients must have the same value. In a high-dimensional problem with many irrelevant SNPs, the benefit of this bias is often greater than the potential disadvantage of obtaining biased (or fused) results, if the appropriate amount of bias is introduced as determined by the regularization parameters. Just as lasso achieves a sparsity bias of the regression coefficients through the 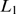 penalty, the fusion penalty plays the role of achieving another type of bias, the sparsity in the *differences* of regression coefficients, by combining information among multiple correlated traits according to the topology of the QTN. As we demonstrate in our simulation study, in a typical association study that involves many irrelevant SNPs, this bias towards sparsity in the *difference* of regression coefficients for neighboring traits helps increase power while reducing false positives, since the information of a SNP being relevant or irrelevant is shared across traits. A balance among the three terms in Eqn 4 that jointly define the objective function, the regression error, the sparsity penalty, and the fusion penalty, will be reached if the optimal regularization parameters 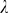 and 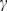 are used when estimating 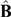. As we describe in the next section, such regularization parameters can be chosen automatically through cross-validation.

### Model II: 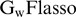


Now, we describe an enhanced version of 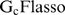, the 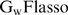, which exploits not only the graph topology of a QTN, but also the edge weights thereof. The 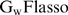 method weights each term in the fusion penalty in Eqn 4 by the amount of correlation between the two traits being fused, so that the amount of correlation controls the amount of fusion for each edge. More generally, 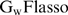 weights each term in the fusion penalty with a monotonically increasing function of the absolute values of correlations, and finds an estimate of the regression coefficients as follows:
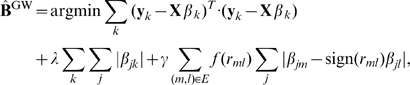
(5)from which the set of QTLs 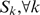 can be uncovered. If the two traits 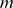 and 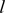 are highly correlated in the QTN 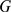 with a relatively large edge weight, the fusion effect over the two traits will intensify, and as a result the difference between the two corresponding regression coefficients 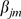 and 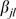 will be penalized more than those for other pairs of traits with weaker correlation. In this article, we consider 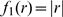 for 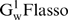 and 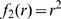 for 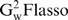. We note that the 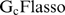 is a special case of the 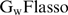 with 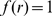.

Compared to 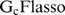, 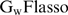 is significantly more flexible due to its usage of the edge weights to incorporate the strength of correlation. For example, when two groups of highly correlated traits show a relatively weaker correlation across the two subnetworks, 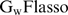 can handle the hierarchical subgroup structure and adjust the amount of fusion accordingly by weighting each fusion term. In addition, when the association strength of a SNP with pleiotropic effect varies over traits in a subnetwork, 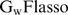 can use different levels of correlations for different pairs of traits to adjust the amount of fusion in 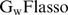. In this case, 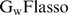 tends to identify multiple blocks of fused regression coefficients within the subnetwork, instead of a single block.

### The Optimization Algorithm

The optimization problems in Eqn 4 and Eqn 5 are convex, and can be formulated as a quadratic programming problem using the similar approach for solving the fused lasso problem [32]. Although there are many publicly available software packages that efficiently solve such quadratic programming problems, these approaches do not scale in terms of computation time to a large problem involving hundreds or thousands of traits as is the case in a typical multiple-trait association study [34]. Since the main difficulty of directly optimizing Eqn 4 and Eqn 5 arises from the non-smooth function of the 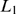 norm, we transform this problem to an equivalent form that involves only smooth functions [35],[36], and use a fast coordinate-descent algorithm to find the estimates of regression coefficients.

In this section, we describe a procedure for obtaining estimates of the regression coefficients in 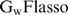. Since 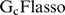 is a special case of 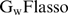 with 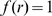, the same procedure can be applied to 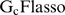 in a straight-forward manner. It can be shown that solving the optimization problem in Eqn 5 is equivalent to solving the following problem with a smooth function of 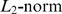 [35],[36]:
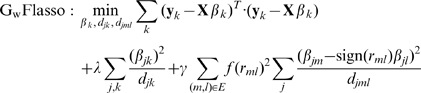
(6)

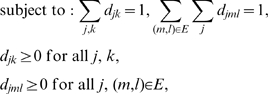
where 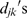 and 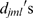 are additional variables that we need to estimate. We solve the above problem using a coordinate-descent approach that iteratively updates variables of interest, 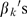, and (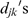, 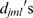), until there is little improvement in the value of the objective function. Using this approach, we first fix values of 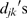 and 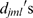, and find the update equation for 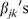 by differentiating the objective function in Eqn 6 with respect to each 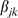 and setting it to 0. The update formula for each 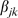 is given as:
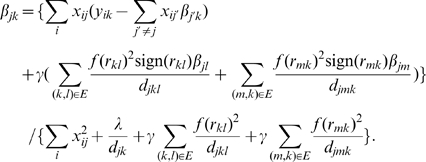
Then, we fix 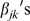, and optimize Eqn 6 over 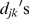 and 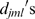 using the following update equations:
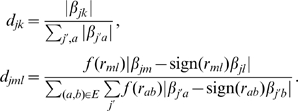



This coordinate-descent procedure finds the optimal 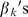 for fixed regularization parameters, 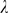 and 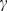. The regularization parameters 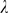 and 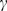 can be determined automatically by a cross-validation or by using a validation set, as was suggested for fused lasso [32]. We divide the dataset into two groups, a training set and a validation set, and estimate the regression coefficients using the training set by running the coordinate-descent procedure on a grid of the regularization parameters 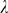 and 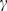, and select the 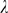 and 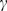 that give the regression coefficients with the lowest squared error on the validation set. Given the regularization parameters that we chose in this manner, we use the combined dataset of both the training and validation sets in order to obtain the final estimate of the regression coefficients.

The coordinate-descent algorithm for 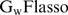 runs reasonably fast for fixed 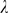 and 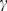, but for a large problem, this type of grid search can be time-consuming. In order to improve the efficiency in computation time, we take a gradient-descent approach that iteratively updates 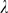 and 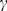 until we reach convergence with little additional improvement in the cross-validation error 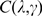 as we describe below. Given the values of the regularization parameters at the 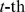 iteration 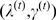, we obtain 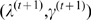 as follows:
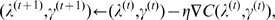
where the gradient 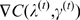 is approximated by a finite difference vector
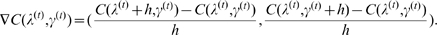
The term 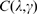 in the above equation can be evaluated by solving Eqn 6 with the given 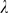 and 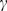.

**Figure 3:**
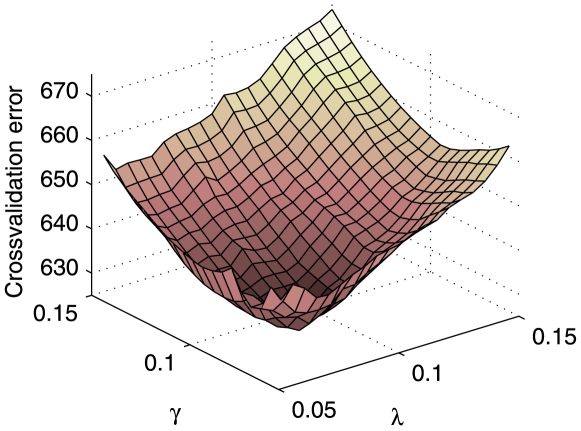
Cross-validation error surface over a grid of regularization parameters (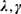) from 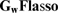. Our goal is to find values for 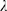 and 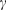 that give the lowest cross-validation error. We use a gradient-descent type of search algorithm to explore this surface of cross-validation error.

We determine the initial values 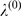 and 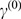 for the gradient descent as follows. We first search for 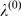 that produces the minimum cross-validation error by solving lasso with 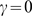. Then, we fix 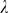 at 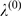, and perform another one-dimensional search in the direction of 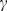, starting from 0 to find the optimal 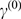 for 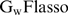 along this path. In our experiments, we found that the initial values obtained by this procedure was sufficiently close to the global optimum, and that it converged to the optimum within a relatively small number of iterations. Figure 3 shows a typical example of cross-validation errors over the grid of 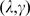 from 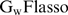. In our experiments, we found that our gradient-descent type of method converged roughly to the same values for the 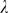 and 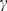 as were selected by the grid search method.

## Results

### Simulation Study

We performed a simulation study to evaluate the power of the proposed GFlasso methods, and compared the results with those from single-marker/single-trait regression analyses as well as other multivariate regression methods.

We simulated genotype data of 50 SNPs for 250 individuals based on the HapMap data [15] in the region of 8.79–9.20 M in chromosome 7. The first 60 individuals came from the parents of the HapMap CEU panel. We generated genotypes for additional 190 individuals by randomly mating the original 60 individuals from the CEU panel. Since our primary goal was to evaluate the advantage of exploiting correlation among multiple traits by using GFlasso, we sampled 50 SNPs randomly from the 697 SNPs in the region in order to reduce the correlation among SNPs from the linkage disequilibrium (LD). We included only those SNPs with minor allele frequencies greater than 0.1.

Given the simulated genotype, we set the number of phenotypes to 10, and simulated the matrix of true regression coefficients by first choosing SNP-trait pairs with true associations and assigning values for the strengths of associations for the selected pairs as we describe below. We assumed three groups of correlated traits of sizes 3, 3, and 4. Three causal SNPs were randomly selected for the first group of traits, and four causal SNPs were selected for each of the other two groups, so that the shared relevant SNPs induce correlation among the traits within each cluster. In addition, we assumed another causal SNP for traits in both of the first two clusters in order to model the situation of a higher-level correlation structure across two subnetworks. Finally, we assumed one additional causal SNP for all of the phenotypes. In our simulation study, we assumed that shared causal SNPs are the only factors that induce correlations among traits, although in general there might be other genotypic effects or environmental factors that influence the correlation structure among traits.

Once the SNP-trait pairs with true association were selected, we considered the following two cases of association strengths for these pairs, while setting the rest of the regression coefficients to 0.

#### • Case 1

The regression coefficients for all of the SNP-trait pairs with true association were set to the same value. This corresponds to the situation where the basic assumption of the fusion penalty holds, and each SNP has the same effect across the traits in each subnetwork.

#### • Case 2

The regression coefficients for the SNP-trait pairs with true associations were set to different values randomly generated from a uniform distribution over an interval 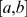. Here, our goal is to see whether the GFlasso methods have the flexibility to adjust the effect of fusion penalty in order to introduce an appropriate amount of bias without sacrificing the power.

Then, we simulated phenotype data based on the linear regression model with noise distributed as 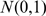, using the simulated genotypes as covariates.

We compared the results from the GFlasso methods with those from other methods given below:

#### • Single-SNP/single-trait regression analysis

We used (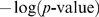) for each SNP-trait pair as a measure of strength of association.

#### • Regularized multivariate regression methods for a single output such as ridge regression and lasso

These methods do not take into account the correlation structure in traits. We used a validation set to select the regularization parameter. The absolute values of the regression coefficients were used as a measure of association strength.

#### • PCA-based regression method for taking into account trait correlations

This method first transforms the output variables (traits) into a smaller number of variables that explain most of the variability in the original data, performs a standard multivariate regression on each of the transformed output separately, and then transforms the estimated regression coefficients back into the original space [17],[18]. Although it considers the trait correlation structure through PCA, the structural information in this approach is less explicit than in the GFlasso methods. We used lasso as a sparse multivariate regression method in the transformed output space. Again, the absolute values of the regression coefficients were used as a measure of association strength.

For methods that require a specification of the values of the regularization parameters such as ridge regression, lasso, and the GFlasso methods, we used 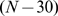 samples out of the total 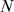 samples as a training set, and the remaining 30 samples as a validation set. Once we determined the regularization parameters, we used the entire dataset of size 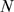 to estimate the final regression coefficients given the selected regularization parameters.

**Figure 4:**
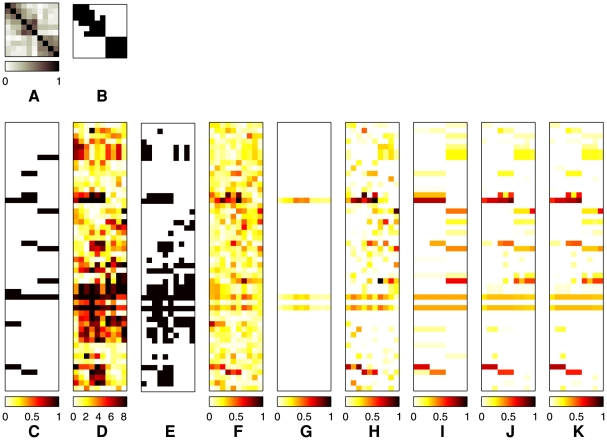
Results of association analysis by different methods based on a single simulated dataset. Association strength 0.8 and threshold 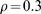 for the QTN were used. (A) The 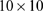 correlation coefficient matrix of traits. It contains three blocks of correlated traits of sizes 3, 3, and 4, respectively. (B) The correlation coefficient matrix in (A) thresholded at 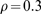. The black pixels in the lower triangular part of the matrix indicate edges included in GFlasso. (C) The true regression coefficients and sparsity pattern used in simulation. (D) 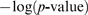, where 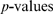 were obtained from single-SNP permutation tests performed for each phenotype separately. (E) Black pixels indicate SNP-trait pairs with significant association at 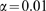 based on the results of 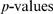 in (D). Values of the estimated regression coefficients are shown for (F) ridge regression, (G) PCA-based regression, (H) lasso, (I) 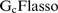, (J) 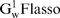, and (K) 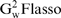. In Panels (C)–(K), rows correspond to SNPs, and columns to phenotypes. Columns for traits in (C)–(K) are aligned with the columns in (A) and (B).

As an illustrative example of the behaviors of the different methods, a graphical display of the QTN and the estimated QTL sets 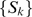 for all 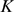 traits in the QTN is presented in Figure 4 for a simulated dataset of 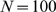 samples, with association strengths (i.e., regression coefficients 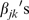) all set to 0.8 for SNP-trait pairs with true associations (Case 1). The 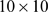 trait correlation matrix in Figure 4A shows blocks of correlated traits. Using a threshold 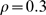, we obtained a QTN in Figure 4B, where the black pixels in the lower triangular part indicate the presence of edges between two traits. Given the true regression coefficients in Figure 4C, we recovered the SNP-trait pairs with true association using our methods and competing ones mentioned above. It is apparent from Figure 4 that many false positives show up in the results of the single-marker/single-trait analyses, multivariate regression methods, and the PCA-based method. Furthermore, these reference benches do not identify the block structure of SNPs affecting multiple traits jointly, which is clear in the true regression coefficients. On the other hand, the results from 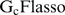 in Figures 4I–K show fewer false positives, and reveal clear block structures. This experiment suggests that borrowing information across correlated traits in a QTN, as in the GFlasso methods, can significantly increase the power of discovering true causal SNPs. Since 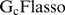 uses an unweighted trait network, often the regression coefficients for a given SNP have been fused excessively across traits even between only weakly correlated traits, especially among the first six traits on the upper left corner of Figure 4B that involve two smaller subnetworks within the subnetwork. This undesirable property of 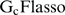 mostly disappeared when we incorporated the edge weights in 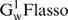 and 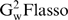 as shown in Figure 4J and Figure 4K.

Next, we systematically and quantitatively evaluated the performance of the association methods based on two criteria, sensitivity/specificity on the uncovered QTL sets 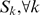, and the trait prediction error. The sensitivity and specificity measure whether the given method can successfully detect the truly associated SNPs with low false positives. The 1-specificity and sensitivity are equivalent to type I error rate and 1-type II error rate, respectively, and their plot is widely known as a receiver operating characteristic (ROC) curve. Once we identify causal SNPs for a trait related to disease susceptibility, we may want to use this information to predict whether a new individual possessing the particular allele at these causal SNP loci has an increased risk for the disease. The trait prediction error measures the accuracy of this prediction by evaluating the results of association analysis on a new set of previously unseen individuals. In order to compute the prediction error in our simulation study, we generated an additional dataset of 50 individuals, 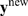 and 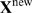, and computed the phenotype prediction error as the sum of squared differences between the true values 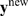 and predicted values 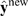 of the phenotypes, 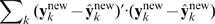, where 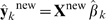. For both criteria for measuring performance, we computed results averaged over 50 randomly generated datasets. Below, we report the performance of GFlasso under a wide spectrum of test conditions likely to be encountered in a realistic genome-wide association analysis of a QTN.

### Varying Sample Sizes

**Figure 5:**
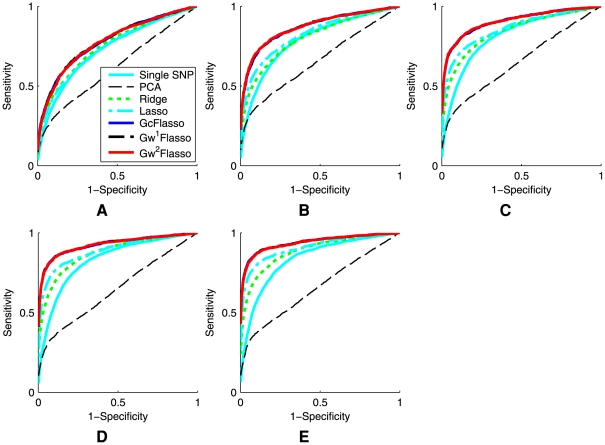
ROC curves comparing the performance of association analysis methods when the sample size varies. Panels show (A) 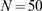, (B) 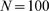, (C) 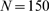, (D) 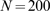, and (E) 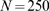. The association strength was 0.5, and the threshold 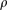 for producing the QTN was set to 0.3. The results were averaged over 50 simulated datasets. The ROC curves for 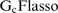, 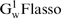, and 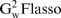 almost entirely overlap.

First, we varied the sample size of the dataset to see how the sample size affects the performance of the different methods for association analysis. We used datasets of sizes 50, 100, 150, 200, and 250, with association strength fixed at 0.5 for all associated SNP-trait pairs (Case 1), and we set the threshold 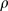 for trait correlations to be 0.3 to learn the QTN. The results are summarized in Figure 5, where the ROC curves were averaged over 50 datasets. The results confirmed that the lasso-based methods such as lasso and GFlasso methods are more successful in identifying true associations than the other methods. In addition, it can be seen that the ROC curves for 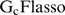, 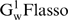, and 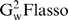 almost entirely overlap, whereas other methods are significantly inferior. We found that across all sample sizes, including a graph-guided fusion penalty as in GFlasso to take advantage of the correlation structure in traits can significantly increase the power for detecting true associations while reducing false positives, compared to lasso and other methods.

### Varying Signal-to-Noise Ratios

**Figure 6:**
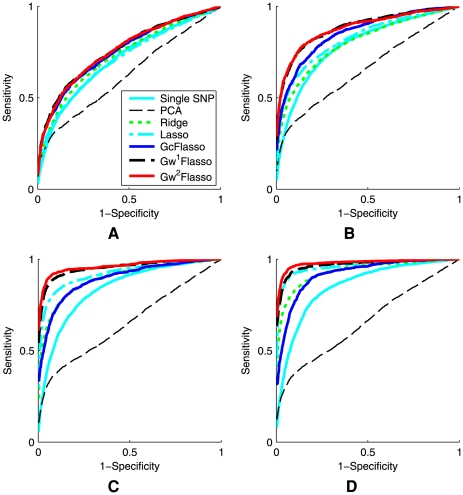
ROC curves comparing the performance of association analysis methods when the association strength varies. Panels show results for association strength (A) 0.3, (B) 0.5, (C) 0.8, and (D) 1.0. The sample size was 100, and the threshold 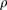 for producing the QTN was set to 0.1. The results were averaged over 50 simulated datasets. In Panel (A), the ROC curves for 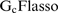, 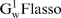 and 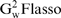 almost entirely overlap. In Panel (B), the ROC curves for 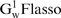 and 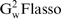 almost entirely overlap.

We examined how varying the signal-to-noise ratio affects the performances of the different methods. We simulated datasets with regression coefficients set to 0.3, 0.5, 0.8, and 1.0, respectively, with sample size 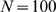. For each dataset, we set the values of the regression coefficients to the same value (again, Case 1). A threshold of 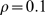 was used to generate trait correlation networks. We applied our methods and the other benchmark methods to recover the SNP-trait pairs with true associations. The resulting ROC curves averaged over 50 datasets are shown in Figure 6. It can be seen that the lasso-based methods have a greater power with fewer false positives than the other methods for all of the different signal-to-noise ratios. Among the GFlasso methods, 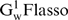 and 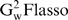 outperformed the other methods for all of the four chosen association strengths. However, the performance of 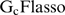 was significantly compromised and became worse than the standard lasso when the association strength was set to high values of 0.8 and 1.0. This is because at the relatively low threshold 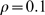, the QTN contained many edges between pairs of traits that were only weakly correlated, and 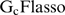 with unweighted fusion penalty did not distinguish edges for strong correlation from those for weak correlation. In contrast, 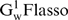 and 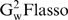 had the flexibility to handle different strengths of trait correlations in the QTN through a weighted fusion penalty, and consistently outperformed the other methods.

### Varying QTN Generation Schemes

**Figure 7:**
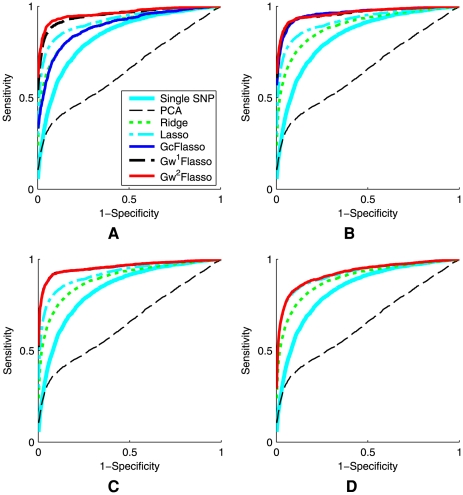
ROC curves comparing association analysis methods when the threshold 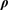 for producing the QTN varies. Panels show the threshold (A) 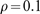, (B) 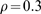, (C) 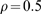, and (D) 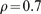. The sample size was 100, and the association strength was 0.8. The results were averaged over 50 simulated datasets. In Panels (B) and (C), the ROC curves for 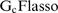, 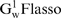 and 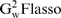 almost entirely overlap. In Panel (D), the ROC curves for lasso, 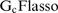, 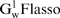 and 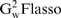 almost entirely overlap.

Next, we examined the sensitivity of the GFlasso methods to how the trait correlation network is generated, by varying the threshold 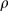 of edge weights from 0.1 to 0.3, 0.5 and 0.7. With lower values of 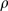, more edges would be included in the QTN, some of which represent only weak correlations. The purpose of this experiment was to see whether the performance of the GFlasso methods is negatively affected by the presence of these weak and possibly spurious edges that were included due to noise rather than from a true correlation. The results for QTL recovery averaged over 50 datasets with sample size 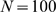 and association strength 0.8 (Case 1), are presented in Figure 7. We also include the ROC curves for the methods that did not use the QTN in each panel of Figure 7 for the ease of comparison. As in Figure 6, 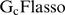 did not have the flexibility of accommodating edges of varying correlation strength in the QTN, and again, this deficiency compromised the performance of 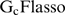 at the low threshold 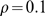, as shown in Figure 7A. On the other hand, 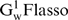 and 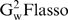 exhibited a greater power than all other methods even at a low threshold 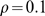. As the threshold 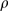 increased, the inferred QTN included only those edges with significant correlations. Thus, the performance of 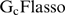 approached that of 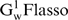 and 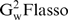, and the ROC curves of the three methods in the GFlasso family overlapped almost entirely (Figure 7B and Figure 7C). When the threshold became even higher, e.g., 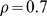, the number of edges in the QTN became close to 0, effectively removing the fusion penalty. As a result, the performances of all of the graph-guided methods approached that of lasso, and the four ROC curves became overlapping (Figure 7D). Overall, we conclude that when flexible structured methods such as 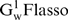 and 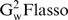 are used, taking into account the correlation structure in phenotypes improves the power of detecting true causal SNPs regardless of the values for 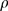. In addition, once the QTN contains edges that capture strong correlations, including more edges beyond this point by further lowering the threshold 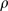 does not significantly affect the performance of 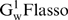 and 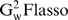.

**Figure 8:**
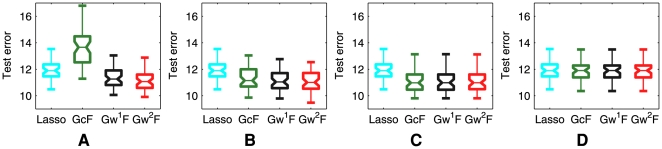
Comparison of association analysis methods in terms of phenotype prediction error. Panels show the prediction errors when the threshold 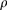 for producing the QTN is (A) 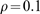, (B) 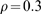, (C) 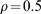, and (D) 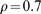. The results were averaged over 50 simulated datasets. The box in each box plot shows the lower quartile, median, and upper quartile values, and the whiskers show the range of the prediction errors in the 50 simulated datasets.

Given the SNP-trait pairs that the association methods found as associated, and the corresponding regression coefficients, we computed prediction errors to see if these SNPs with non-zero regression coefficients had a predictive power for traits of previously unseen individuals. Figure 8 shows the trait prediction error using the model learned from the above experiments summarized in Figure 7. It can be seen that 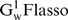 and 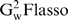 generally offer a better predictive power than other methods, except for the case where the set of edges for the QTN becomes nearly empty due to the high correlation threshold 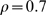 (Figure 8D). In this case, all of the GFlasso methods and lasso performed similarly.

### Variable Association Strength between a SNP and Correlated Traits

**Figure 9:**
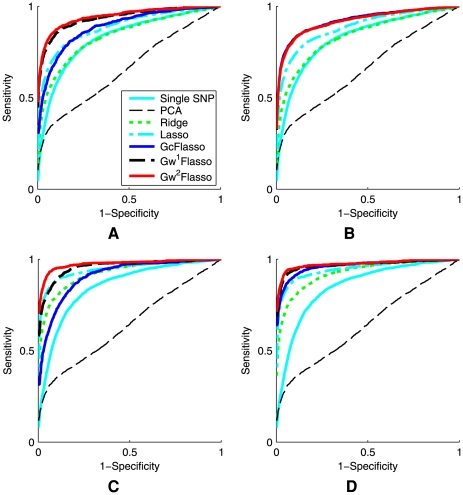
ROC curves comparing association analysis methods. The association strength of a causal SNP is not uniform across correlated phenotypes that the SNP is associated with, and varies within the intervals of [0.3, 0.6] or [0.6, 0.9]. Panels show (A) association strength = [0.3, 0.6] when the threshold 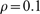 is used for QTNs, (B) association strength = [0.3, 0.6] when 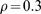, (C) association strength = [0.6, 0.9] when 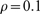, and (D) association strength = [0.6, 0.9] when 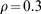. The sample size was 200. The results were averaged over 50 simulated datasets.

Since the fusion penalty tends to fuse the regression coefficients to be the same value within a densely connected subgraph, one may suspect that the bias introduced by this penalty can reduce the power when the true association strengths of a SNP to different traits are not the same within each subgraph. In order to examine how the performance is affected in this case, we considered the situation where the association strengths of each causal SNP are not uniform across traits within each subnetwork, but vary within the interval of 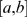 (Case 2). We experimented with two different intervals [0.3, 0.6] and [0.6, 0.9], and summarized the results in Figure 9. Sample size 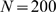 with thresholds 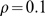 and 0.3 were used, and the ROC curves were averaged over the 50 datasets. We found that 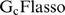 sometimes performed worse than lasso that does not take into account the trait correlation structure, as can be seen in Figure 9C. However, 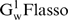 and 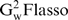 remained dominating over the other methods. Our results suggest that with the flexibility of the weighted fusion penalty as in 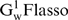 and 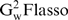, the benefit of borrowing information across correlated traits outweighes the adverse effect of encouraging the regression coefficients to be fused even when their values are not the same.

### Computation Time and Scalability

**Figure 10:**
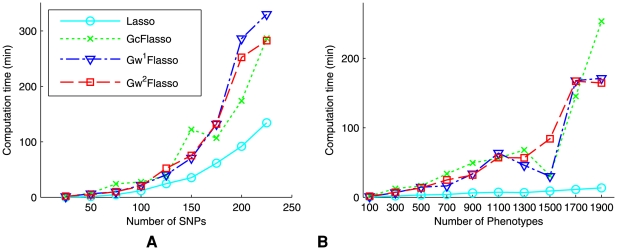
Comparison of the computation time for lasso, 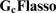, 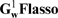, and 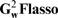. (A) We varied the number of SNPs with the number of phenotypes fixed at 250. (B) We varied the number of phenotypes with the number of SNPs fixed at 50. The QTNs were obtained using threshold 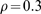. The number of edges in the QTNs ranged from 900 to 950 in each case.

The scalability of our methods can be assessed from Figure 10, where the computation time for solving the optimization problem for lasso, 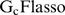, 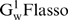, and 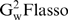 with fixed regularization parameters is shown. In Figure 10A, the number of traits in the QTN was fixed at 250, and the number of SNPs varied over the illustrated range. With 100 SNPs and 250 traits, the running time was around 20 minutes for the GFlasso methods, suggesting that a sliding-window scheme along the genome would be more reasonable for a whole-genome scan than considering all of the SNPs in a single model. Figure 10B shows the time cost over varying number of traits, with the total number of SNPs fixed at 50. We found that the GFlasso methods could handle at least hundreds of traits reasonably well. For a large dataset with more than several thousand traits, one might consider first breaking down the whole network into smaller components and then running GFlasso on each component separately.

### Association Analysis of Polymorphisms in *IL-4R* Gene and Severe-Asthma Traits

We applied our methods to a dataset collected from 543 asthma patients as a part of the Severe Asthma Research Program (SARP) [14]. The genotype data were obtained for 34 SNPs within or near the *IL-4R* gene that spans a 40 kb region on chromosome 16. This gene has been previously shown to be implicated in severe asthma [37]. We used the publicly available software *PHASE* [38] to impute missing alleles and phase the genotypes. The phenotype data included 53 clinical traits related to severe asthma such as age of onset, family history, and severity of various symptoms. Our goal was to examine whether any of the SNPs in the *IL-4R* gene were associated with a subnetwork of correlated traits rather than an individual trait. We standardized measurements for each phenotype to have mean 0 and standard deviation 1 so that their values were roughly in the same range across phenotypes.

**Figure 11:**
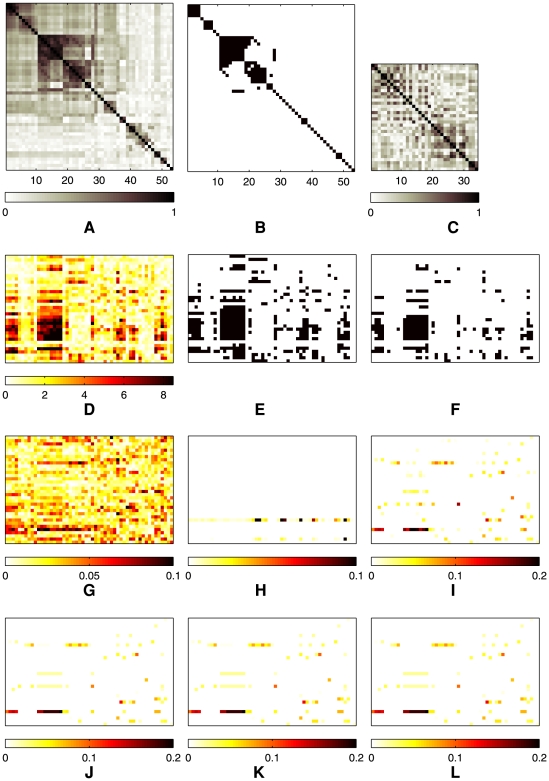
Results from the association analysis of the asthma dataset. (A) The correlation matrix of 53 asthma-related clinical traits. A pixel at row 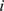 and column 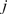 corresponds to the absolute magnitude of correlation between node 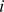 and 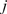 in the QTN depicted in Figure 1. (B) The trait correlation matrix thresholded at 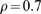. The black pixels in the lower triangular part of the matrix indicate edges between each pair of traits. (C) The matrix of 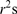 shows the linkage disequilibrium structure in the 34 SNPs in gene *IL-4R*. (D) 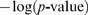 from single-marker/single-trait association tests after 2000 permutations. (E) The SNP-trait pairs that the single-marker/single-trait analyses with permutation tests in (D) find significant at 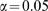 are shown as black pixels. (F) The SNP-trait pairs with significant association at 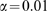 based on the 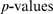 in (D) are shown as black pixels. Estimated 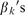 are shown for (G) ridge regression, (H) PCA-based regression, (I) lasso, (J) 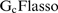, (K) 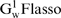, and (L) 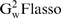. In Panels (D)–(L), rows correspond to SNPs, and columns to phenotypes.

Before searching for associations between SNPs and traits, we first examined the correlation structure in the 53 clinical traits in question. We first computed the pairwise correlations between these traits as depicted in Figure 11A, and thresholded the correlations at 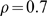 to obtain the QTN in Figure 1. The rows and columns in the matrix in Figure 11A were ordered via an agglomerative hierarchical clustering algorithm so that highly correlated traits were next to each other in the linear ordering and formed apparent blocks in the matrix corresponding to subsets of highly inter-correlated traits. Recall that 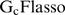 uses only edge connectivities but not their weights in the QTN. For the ease of comparison, we graphically display this QTN in Figure 11B, where the black pixel at position 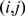 indicates that the 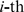 and 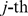 phenotypes are connected with an edge in the QTN. It is easy to see the correspondences between the blocks (i.e., clusters) of black pixels in Figure 11B and the subgraphs of correlated traits in Figure 1. For example, the traits representing quality of life of the patients (the nodes for QLEnvironment, QLSymptom, QLEmotion, and QLActivity) appear as a small subnetwork near the center of Figure 1 as well as the block of black pixels at the upper left corner of Figure 11B. We find another subnetwork consisting of three traits related to asthma symptoms (the nodes for Wheezy, Sputum, ChestTight) near the upper right corner of Figure 1 and as the second cluster from the left in Figure 11B. The cluster of traits from columns 11 through 18 and the next cluster from columns 19 through 25 in Figure 11B corresponds to the two densely connected subnetworks within the large subnetwork on the left-hand side of Figure 1 that consists of traits related to lung physiology (the nodes for BaseFEV1, PreFEFPred, PostbroPred, PredrugFEV1P, MaxFEV1P, etc.). Based on Figure 1 and Figure 11B, we concluded that the QTN obtained at threshold 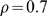 captured the previously known clusters of asthma-related traits, and we used this network in our multiple-trait association analysis with GFlasso methods.

A comprehensive comparison of QTL mapping using GFlasso and other methods is presented in Figures 11D–11L, of which each panel displays the matrix of estimated association strengths of all marker genotypes versus all phenotypic traits. The rows and columns represent genotypes and phenotypes, respectively. The phenotypes in the columns are ordered in the same way as in Figure 11A and Figure 11B. We first performed a baseline single-marker/single-trait pairwise association analysis with a permutation test, and obtained 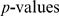 after 5000 permutations. The 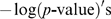 are shown in Figure 11D. Based on these 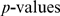, the SNP-trait pairs significant at 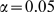 and 0.01 are shown as black pixels in Figure 11E and Figure 11F. The strengths of associations found by the six different multivariate regression methods including ridge regression, PCA-based method, lasso, 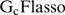, 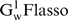, and 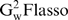 are shown in Figures 11G–L, respectively. We selected the regularization parameters in lasso, 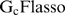, 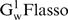, and 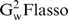 using a five-fold cross validation. For all of these methods, we used the absolute values of the estimated regression coefficients as a measure of association strength.

As can be seen from Figure 11, all of the methods for association analysis except for the PCA-based one in Figure 11H found the SNP in row 30 near the bottom, known as Q551R, as significantly associated with a block of correlated phenotypes in columns 11–18 of Figure 11A that are related to lung physiology (consisting of BaseFEV1, PreFEFPred, AvgNO, BMI, PostbroPred, BaseFEVPer, PredrugFEV1P, MaxFEV1P, FEV1Diff, and PostFEF). In particular, the 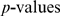 for this SNP across this block of traits from the single-marker analyses were 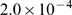. This SNP Q551R resides in exon 12 of gene *IL-4R*, and codes for amino-acid changes in the intracellular signaling portion of the receptor. It has been previously found to be associated with severe asthma and its traits for lung physiology [37], and our results confirmed this previous finding.

In addition, the results from the single-marker analyses in Figures 11D–F showed that on the upstream of SNP Q551R, there was a set of adjacent SNPs (rows 24–27) that had generally a high level of association with the same subset of traits for lung-physiology with 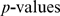 ranging from 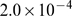 to 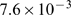. In contrast, lasso set the regression coefficients for most of this block of SNPs to zero (Figure 11I). When we examined the LD structure in this region as shown in Figure 11C, we found that the SNPs in rows 26 and 27 were in a strong LD with SNP Q551R (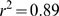 and 0.76, respectively). Thus, lasso was able to ignore the possibly irrelevant markers (rows 26 and 27) that are merely in a strong LD with the causal SNP (SNP Q551R) by setting the corresponding regression coefficients to zero. This confirmed that lasso is an effective method for finding the sparse structure in regression coefficients. On the other hand, the other two SNPs in the same block in rows 24 and 25 were in a weak LD with SNP Q551R (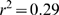 and 0.42, respectively). This suggests that these two SNPs might be unknown causal SNPs that lasso missed because of its property of favoring sparsity. The results from ridge regression as shown in Figure 11G did not show a sparse structure as in the lasso estimates. In fact, in statistical literature, it is well-known that ridge regression performs poorly in problems that require a selection of a small number of markers affecting phenotypes, compared to lasso. Since the methods in the GFlasso family include the lasso penalty, the results from 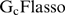, 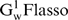, and 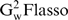 in Figures 11J–L showed the same property of sparsity as lasso in their estimates, and the regression coefficients corresponding to the SNPs in rows 24–27 and lung-physiology traits were set to zero.

Because of the fusion penalty, the regression coefficients estimated by our methods formed a block structure, where each block corresponds to a SNP associated with several correlated traits. It is clear that the horizontal bars in Figures 11J–L are generally aligned with the blocks of highly correlated traits in Figure 11A. Although the fusion penalty tends to fuse the values of regression coefficients for each SNP across correlated traits to the same value, each horizontal bar does not always necessarily consist of regression coefficients of the same value, but often contain several small blocks of fused values. This is because the fusion penalty only introduces bias towards a shared association strength of relevant SNPs among correlated traits with the flexibility of adapting to the data rather than being a hard constraint. The same block structure was much weaker in the results from lasso shown in Figure 11I. For example, Figures 11J–L show that SNPs rs3024660 (row 22) and rs3024622 (row 18) on the upstream of SNP Q551R are associated with the same block of traits as SNP Q551R, generating an interesting new hypothesis that these two SNPs as well as SNP Q551R might be jointly associated with the same subset of traits for lung physiology. Although the single-marker/single-trait analyses also found these two SNPs reasonably significant (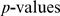 of SNP rs3024660 in the range of 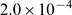 and 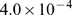, and SNP rs3024622 in the range of 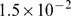 and 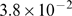 across the traits for lung physiology), the results were more noisy with many positives for SNPs in LD such as SNPs in rows 20–24. Also, this block structure shared by these two SNPs and SNP Q551R was not obvious in the results of the other multivariate regression methods that analyzed each trait separately.

In order to see how the threshold 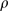 for creating the QTN affects the results, we fit lasso and our methods in the GFlasso family for different values of 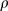, and summarized the results in Table 1. When the threshold was high at 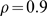, only a very small number of edges were included in the QTN, and the graph-guided fusion penalty of GFlasso had little effect. Thus, the number of non-zero regression coefficients found by 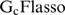, 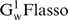, and 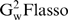 was similar to the result of lasso that does not have a fusion penalty. When we lowered the threshold to 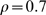, the number of non-zero regression coefficients decreased significantly for the GFlasso methods. However, as we further lowered the threshold, the number of non-zero regression coefficients generally remained unchanged. This is because most of the significant correlation structure was captured in the QTN at 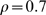 as can be seen in Figure 11B. Adding more edges by further lowering 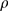 did not add any significant correlation information to the QTN, and the results of the GFlasso methods were not sensitive to these additional edges with relatively little information.

**Table 1:** Summary of results for the association analysis of the asthma dataset.

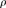	Number of edges	Number of nonzero regression coefficients			
		Lasso	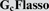	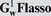	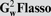
0.3	421		105	106	108
0.5	165	125	108	107	107
0.7	71		105	105	110
0.9	11		125	123	123

In summary, the GFlasso methods identified the previously known causal SNP (SNP Q551R) as significantly associated with the lung physiology traits, while maintaining an overall sparse pattern in estimated regression coefficients to reduce false positives. The property of the GFlasso estimates having a block structure for a SNP jointly associated with a set of correlated traits led to an interesting new hypothesis that two additional SNPs (rs3024660 and rs3024622) on the upstream of SNP Q551R may be jointly influencing the same set of traits on lung physiology as SNP Q551R, which may be validated in a future follow-up study.

## Discussion

When multiple phenotypes are involved in association mapping, it is important to combine the information across phenotypes and make use of the full information available in data in order to achieve the maximum power. Most of the previous approaches either considered each phenotype separately, or used relatively primitive types of phenotype correlation structures such as surrogate phenotypes transformed through PCA or the mean values of subgroups of phenotypes found by clustering algorithms. Networks or graphs have been extensively studied as a representation of the correlation structure of phenotypes such as gene expression or clinical traits because they provide a flexible and explicit form of representation for capturing dependencies [39]–[41]. A QTN contains rich information on phenotype interaction patterns such as densely connected subgraphs that can be interpreted as a cluster of phenotypes participating in the same biological process. Developing a tool for multiple-phenotype association mapping that can directly leverage this full graph structure of a QTN can offer a way to combine the large body of previous research in network analysis with the work on association mapping.

In this article, we proposed a new family of regression methods called GFlasso that directly incorporates the correlation structure represented as a QTN and uses this information to guide the estimation process. These methods considered a multitude of phenotypes jointly, and estimated a joint association model in a single statistical framework. Often, we are interested in detecting genetic variations that perturb a sub-module of phenotypes rather than a single phenotype, and GFlasso achieved this through a fusion penalty, in addition to the lasso penalty, that encourages parsimony in the estimated model. The fusion penalty locally fused two regression coefficients for a pair of correlated phenotypes, and this effect propagated through edges of the QTN, effectively applying fusion to all of the phenotypes within each subgraph. 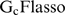 used an unweighted graph structure as a guide to find a subset of relevant covariates that jointly affect highly correlated outputs, whereas 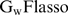 used additional information of edge weights to further control the coupling among phenotypes. Using simulated and asthma datasets, we demonstrated that including richer information on phenotype structure as in 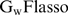 and 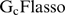 improves the accuracy in detecting true associations.

The fusion penalty in GFlasso introduced a bias that the amount of influence of a shared QTL is similar over the set of correlated traits in order to increase the power for detecting weak signal and reduce false positives. The simulation results showed that the benefit of information sharing due to the fusion penalty outweighed the risk of low-variance bias on fused regression coefficients when in reality the magnitudes of the coefficients can be highly variable. Perhaps a more effective approach and a promising future direction would be to encourage each SNP marker to be jointly relevant or irrelevant to the subset of correlated traits, but still allow the marker to have a different amount of influence on each of the traits. This would reduce the bias introduced by the fusion penalty and further improve the performance of GFlasso, since the only information shared across correlated traits is the sparsity pattern but not the magnitudes of the regression coefficients.

We have used a simple scheme of a thresholded correlation graph for learning the QTN of phenotypes to be used in GFlasso. Many different types of network-learning algorithms have been developed previously. For example, graphical Gaussian models (GGMs) [42] were constructed based on partial correlations that capture the direct influence of interacting nodes, and have been commonly used for inferring gene networks from microarray data [43]. Furthermore, in order to handle the case of a large number of nodes and a relatively small sample size, methods for estimating sparse GGMs have been developed [44]. It would be interesting to see if using more sophisticated graph learning algorithms can improve the performance of GFlasso.

In this study, we assumed that the graph structure of a QTN is available from a pre-processing step. One of the possible extensions of the proposed method is to learn the QTN and the regression coefficients jointly by combining GFlasso with the graphical lasso [45] that learns a sparse covariance matrix for phenotypes. In *Geronemo* and *Lirnet*, both the module network structure and the markers of regulators regulating the modules were learned simultaneously, although these methods only focused on modeling the relationship between regulators and target genes [9],[20]. Extending GFlasso to learn both the graph structure and regression coefficients jointly may further increase the power in an association analysis.

For any new multivariate genetic-association methods, a natural question is whether the new method can scale to a genome-scale analysis. The current implementation of GFlasso leaves this to be determined by a user-specified tradeoffs between power and computation time. As shown in Figure 10, the larger the number of traits and genotypes to be modeled jointly, naturally the greater the computational cost. Thus, users are offered a wide range of tradeoff between computation time and power of the analysis, from single-marker/single-trait per test as in the conversional analysis, to 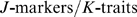 per test with our methods still at a reasonable time (comparable to the time cost of standard lasso), where 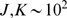. Therefore, instead of scanning the whole genome one marker at a time for each trait separately as in a classical analysis, with our method, one can scan 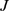 markers at a time using a sliding window for each phenome represented as subnetworks in a QTN. An important future direction is to scale up our methods for even larger values of 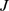 and 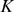, and our proposed graph-guided regression formalism represents a nontrivial and practical initial foray into this direction. We expect that with the development of a new mathematical optimization methodology and faster computing machinery, it will become feasible to handle a wider range of structure sizes based on our model, and a genome-wide association study can depart further away from an unstructured single-marker/single-trait analysis.

Finally, it is important to point out that as of now GFlasso considers only dependencies among phenotypes, and does not assume any dependencies among the markers. Since recombinations break chromosomes during meiosis at non-random sites, segments of chromosomes rather than an individual nucleotide are inherited as a unit from ancestors to descendants, creating a relatively low diversity in observed haplotypes than would be expected if each allele were inherited independently. Thus, SNPs in high LD are likely to be jointly associated with a phenotype in a regression-based penetrance function. In our future research, we plan to apply the same idea of the graph-guided fusion penalty for phenotypes to incorporate the LD structure among genotypes. It is straightforward to introduce another fusion penalty for correlated markers based on the genotype correlation graph and weight each term in the penalty using values that reflect the recombination rates and distances between each pair of genetic markers. This would allow a genome-phenome association analysis for identifying a block of correlated markers influencing a set of correlated phenotypes.

Software for our proposed method is available at http://www.sailing.cs.cmu.edu/gflasso.html. A preliminary version of this method was presented at the 19th international conference on intelligent systems for molecular biology (ISMB 2009).
